# Early Currents: Developmental Electrophysiology and Arrhythmia in Pediatric Congenital Heart Disease

**DOI:** 10.3390/jcdd12100386

**Published:** 2025-10-01

**Authors:** Lixia Dai, Weilin Liu, Vehpi Yildirim, Mathijs S. van Schie, Yannick J. H. J. Taverne, Natasja M. S. de Groot

**Affiliations:** 1 Department of Cardiology, Erasmus Medical Center, 3015 GD Rotterdam, The Netherlands; l.dai@erasmusmc.nl (L.D.); w.liu@erasmusmc.nl (W.L.); v.yildirim@erasmusmc.nl (V.Y.); m.vanschie@erasmusmc.nl (M.S.v.S.); 2 Department of Cardiothoracic Surgery, Erasmus Medical Center, 3015 GD Rotterdam, The Netherlands; y.j.h.j.taverne@erasmusmc.nl; 3 Department of Microelectronics, Signal Processing Systems, Faculty of Electrical Engineering, Mathematics and Computer Sciences, Delft University of Technology, 2628 CD Delft, The Netherlands

**Keywords:** congenital heart disease, pediatric arrhythmia, cardiac electrophysiology, cardiac maturation

## Abstract

Arrhythmias significantly contribute to morbidity and mortality in patients with congenital heart disease (CHD). While postoperative factors predisposing to arrhythmias are well-established, early electrophysiological alterations in pediatric CHD remain poorly understood. This review summarizes current knowledge on postnatal cardiac maturation, conduction-system development, and electrophysiological abnormalities in pediatric patients with and without CHD. Importantly, arrhythmia prevalence, mechanisms, and clinical relevance are systematically discussed across three pediatric groups, including healthy children and patients with unrepaired and repaired CHD. Understanding developmental arrhythmogenic mechanisms may facilitate early risk stratification, guide clinical management decisions, and improve long-term outcomes for pediatric patients with CHD. This review discusses the complex interplay between cardiac maturation, congenital defects, and arrhythmogenesis. It also outlines future directions that include noninvasive monitoring, selective intraoperative mapping, animal model studies, and standardized data collection to improve early risk stratification and long-term outcomes in children with CHD.

## 1. Introduction

Congenital heart disease (CHD) affects approximately 0.8% to 1.2% of live births worldwide [1]. Advancements in surgical repair have significantly improved the long-term prognosis for CHD, with nearly 85% of patients reaching adulthood [1,2,3,4,5]. However, arrhythmias remain a leading cause of morbidity and mortality [6]. While postoperative factors such as surgical scars, residual defects, and altered hemodynamics contribute to arrhythmogenesis [7], the inherent electrophysiological properties predisposing patients with CHD to arrhythmias later in life are less understood.

Postnatal hearts undergo significant remodeling involving morphology, ion channels, and the specific conduction system [8]. However, these developmental processes are frequently disrupted in CHD [9], which may increase vulnerability to arrhythmias.

Emerging evidence, such as conduction abnormalities in pediatric patients with CHD identified by intraoperative epicardial mapping, revealed the existence of electrophysiological alterations early in life [10,11,12]. These alterations could theoretically predispose patients with CHD to arrhythmias as they age. Indeed, long-term follow-up studies have demonstrated that patients with CHD have a higher atrial fibrillation (AF) incidence, earlier AF onset, and increased AF-related mortality rates compared to the general population [9,13,14,15].

Previous studies have predominantly focused on arrhythmogenesis in adult patients with CHD, in whom risk factors such as surgical scars, chronic hemodynamic overload, structural abnormalities, and fibrosis are present [7,16]. These observations raise an essential yet unsolved question: does the CHD heart also differ intrinsically from the normal heart during childhood in a way that makes these individuals more susceptible to arrhythmias later in life, independent of (late) postoperative factors?

To address this gap, this review discusses current knowledge on the development of the heart and its conduction system in both healthy and CHD-affected individuals before adulthood, with emphasis on intrinsic anatomic and electrophysiological differences that may contribute to arrhythmias. We summarize the incidence of the different types of arrhythmias in children with and without CHD, before and after cardiac surgery. Finally, we outline potential future directions to improve early risk stratification and long-term outcomes in the pediatric CHD population.

## 2. Maturation of the Healthy Heart

Postnatal cardiac development involves significant changes in size, cellular composition, and electrophysiological function.

### 2.1. Embryology of the CCS

The cardiac conduction system (CCS) is composed of specialized cardiomyocytes (CMs) responsible for coordinated atrioventricular contraction [17,18]. The CCS, comprising the sinoatrial node (SAN), atrioventricular node (AVN), His bundle, right and left bundle branches, and Purkinje fibers, is largely established by the end of the first trimester [19] and continues to undergo significant structural and functional maturation after birth [19,20].

The CCS develops as specialized tissue by suppression of the default cardiac genetic program for the working myocardium, resulting in reduced cellular proliferation at specific areas in which automaticity is retained. These areas are characterized by poor intercellular coupling [18]. By day 23 of gestation, cells within the primitive heart tube already show automaticity [21,22]. The earliest pacemaker activity emerges at the venous pole, which later gives rise to the SAN [23]. As the endocardial cushions form, the AVN arises in the region just superior and posterior to the cushions within the atrioventricular canal. This location contributes to the functional delay from the atria towards the ventricles [24,25].

In the fetal SAN and AVN, pacemaker cells (P-cells) appear disorganized without distinct clustering [19]. During the first two weeks after birth, they cluster into discrete units as transitional cells increase alongside a progressive collagen framework, reflecting SAN maturation [19]. In the AVN, fetal P-cells aggregate diffusely with irregular branching, which smooths out postnatally [19]. Collagen deposition increases in both nodes after birth, likely playing a role in postnatal morphogenesis [19]. The His bundle, initially extremely large in comparison to the adult heart, also undergoes significant postnatal remodeling [26]. During the first year of life, it appears shaggy but develops into a smoother, more cylindrical structure, which is more pronounced on its left side [19]. These structural changes in the CCS, particularly within the AVN and His bundle during the first year of life, coincide with the typical age range for sudden infant death syndrome (SIDS) [19,27]. This raises the hypothesis that electrical instability of the immature CCS may play a role. Studies have demonstrated a relationship between SIDS and a prolonged QTc [28], and variations in LQTS genes (SCN5A, KCNQ1, KCNH2) have been reported [29,30,31]. A standard surface ECG may help detect QTc prolongation and help prioritize infants for targeted genetic testing with family screening. Prospective longitudinal cohorts with serial ECGs with age- and sex-specific thresholds and integration with genetic testing are needed to improve risk stratification.

### 2.2. Electrophysiological Maturation of the Healthy Heart

Postnatal electrophysiological maturation involves dynamic changes in ion channel expression, localization, function, and action potential characteristics [32,33]. Animal studies have shown that developmental modulation in transient outward potassium currents (*I_to_*), inward rectifier currents (*I_k_*_1_) [34,35,36,37,38,39,40], and T-type calcium currents (*I_Ca_*_,*T*_) [41] contribute to species- and region-specific alterations in action potential duration. In humans, pediatric right atrial appendage CMs have larger *I_to_* currents [42] and faster L-type calcium current (*I_Ca_*_,*L*_) inactivation [43] than adult CMs. Consequently, pediatric atrial action potentials are shorter with a more triangular morphology, contrasting with the prolonged action potential duration with a more prominent notch followed by a longer plateau in the adult right atrial appendage [42,44].

**Figure 1:**
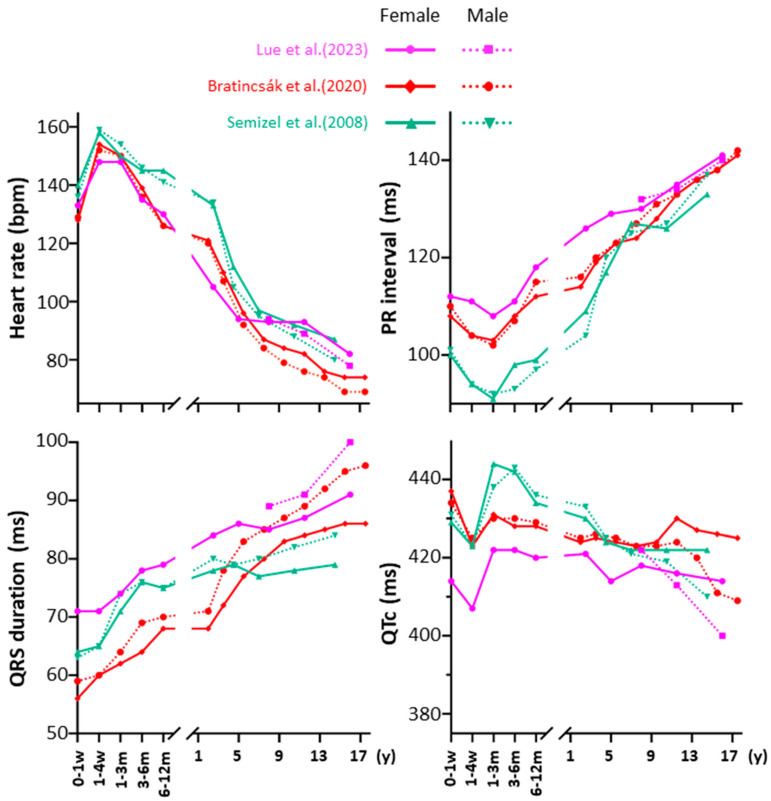
Age-related changes in ECG parameters during development and maturation. Graphs illustrate dynamic trends in key ECG indices from birth to adolescence, including heart rate, PR interval, QRS duration, and QTc interval. Data are stratified by sex to highlight differences between males and females across developmental stages [45,46,47].

Electrophysiological maturation leads to age-dependent changes in the surface electrocardiogram (ECG). In three major studies, collectively including over 30,000 healthy individuals, age- and sex-related trends in ECG parameters have been identified, as summarized in Figure 1 [45,46,47].

Heart rate peaks within the first month of life and gradually declines thereafter. The PR interval shortens at 1–3 months before steadily increasing with age. QRS duration shows a continuous age-related increase, while the QTc interval shortens around one month of age, followed by a transient rise before starting to gradually decline with age. These ECG changes remain consistent across different sexes, ethnicities, and geographic regions. Additionally, newborns typically display right axis deviation due to physiological right ventricular (RV) hypertrophy, which normalizes with age.

## 3. Development of the Pediatric Heart with CHD

CHD has a significant impact on cardiac anatomy and function, including disturbances in the CCS. These alterations collectively establish the foundation for increased susceptibility to arrhythmias in patients with CHD.

### 3.1. CHD-Associated Conduction System Abnormalities

Most CHDs arise during the early phases of embryonic development [48], which may cause structural and/or functional abnormalities of the CCS as well [18,49,50]. As summarized in Table 1, abnormalities in the CCS are closely related to the specific subtype and anatomy of the CHD [7].

AFL: atrial flutter; ASD: atrial septal defect; AVB: atrioventricular block; AVN: atrioventricular node; AVNRT: atrioventricular nodal reentrant tachycardia; AVSD: atrioventricular septal defect; ccTGA: congenitally corrected transposition of the great arteries; CHD: congenital heart disease; pmVSD: perimembranous ventricular septal defect; SAN: sinoatrial node; SND: sinus node dysfunction; SVT: supraventricular tachycardia; VT: ventricular tachycardia.

In atrial septal defects (ASDs), secundum ASDs are generally anatomically remote from the atrioventricular conduction tissue. However, surgical repair of large secundum ASDs can impact the AV nodal fast pathway and potentially predispose patients to atrioventricular nodal reentrant tachycardia (AVNRT) [51].

Higher-grade AV nodal conduction abnormalities are most frequently seen in primum ASDs due to posteroinferior displacement of the AVN and His bundle [51]. AV septal defect (AVSD) [51,54] and perimembranous ventricular septal defect (pmVSD) [52,53] share a similar displacement of the AVN and His bundle. This abnormal positioning predisposes patients with AVSD and pmVSD to an increased risk of postoperative bradyarrhythmia, particularly AV block (AVB). In addition, anatomical abnormalities of the CCS in AVSD include a shortened distance between the AVN and the origin of the left bundle branch, as well as hypoplasia of the left bundle branch [55]. Notably, sinus node dysfunction (SND) may occur in all ASD subtypes both before and after surgery, but it is most common in superior sinus venosus ASDs due to the defect’s close proximity to the SAN, especially following surgical intervention [51].

In contrast, complex CHDs, such as congenitally corrected transposition of the great arteries (ccTGA), atrial isomerism, and Ebstein’s anomaly demonstrate more profound developmental and positional abnormalities of the CCS.

In the normal heart, the compact AVN sits at the apex of the triangle of Koch and gives rise to a His bundle that penetrates the central fibrous body beneath the membranous septum. In ccTGA, AVN is typically antero-superior on the right-sided AV valve near the pulmonary–mitral fibrous continuity, with a long penetrating His bundle that courses anteriorly and leftward in the subpulmonary outflow septum before bifurcating [61,62]. This increases the risk of AV conduction disease, particularly when patients age or undergo surgical repair [56]. The reported incidence of AVB is about 2% per year [61,63], with 10% initially presenting with complete AVB [61]. Given the displacement of the CCS, conventional epicardial LV apical/mid-lateral pacing in pediatric patients [64,65,66,67,68,69,70] is generally not preferred. However, His bundle pacing from the right septal side may reduce ventricular dyssynchrony [61,62].

**Figure 2:**
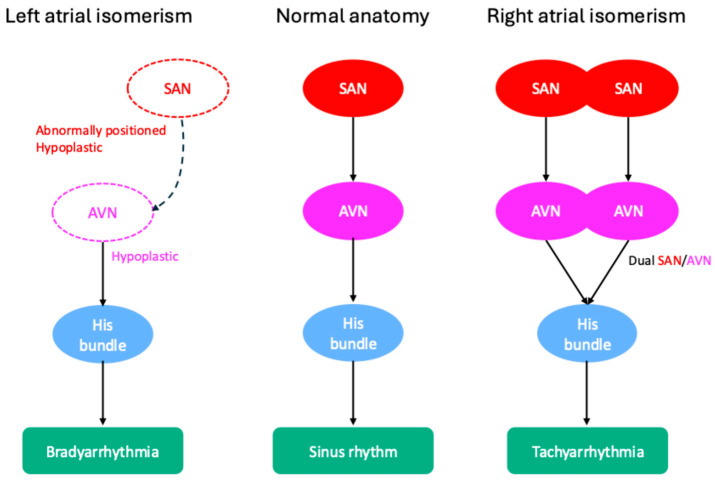
Conduction system variations in left and right isomerism. AVN: atrioventricular node; SAN: sinoatrial node.

Atrial isomerism is associated with subtype-specific abnormalities of the CCS. As demonstrated in Figure 2, in left atrial isomerism, the SAN is often hypoplastic or abnormally positioned, predisposing patients to bradyarrhythmias such as SND and AVB. In contrast, right atrial isomerism may feature duplication of the SAN and AVN, which can create multiple conduction pathways and increase the risk of supraventricular tachyarrhythmias (SVTs) and, less commonly, ventricular tachyarrhythmias [58,59].

Ebstein’s anomaly may involve a compressed AVN and abnormal or fibrosed right bundle branch. Additionally, accessory pathways are often present near the malformed tricuspid valve, predisposing patients to AVB, pre-excitation, and tachyarrhythmias [60].

### 3.2. Epicardial Mapping in Pediatric Patients with CHD 

Postnatal hemodynamic pressure and/or volume overload in CHD may impair normal cardiac development, thereby inducing electrophysiological alterations. However, data on electrophysiological alterations in pediatric patients with CHD is rare. Prior mapping studies have reported on electrophysiological alterations already present during short-term hemodynamic overload in pediatric patients with CHD. These alterations may potentially contribute to the long-term risk of arrhythmias [10].

We recently reported on conduction abnormalities in Bachmann’s Bundle, a preferential pathway for interatrial conduction, in very young pediatric patients with CHD. These early abnormalities may increase the susceptibility of developing atrial tachyarrhythmias later in life [12].

## 4. Pediatric Arrhythmias: From Healthy Hearts to Congenital Defects

Based on the maturation of healthy hearts and the alterations associated with CHD, this section describes arrhythmias in (1) children without CHDs, (2) children with CHDs prior to surgical correction, and (3) those following corrective surgery. While existing reviews predominantly focus on arrhythmias in adult patients with CHD [9,13,16,71], early-onset arrhythmias in childhood are being increasingly recognized. These arrhythmias may be resolved with cardiac maturation, whereas other arrhythmias may persist or progress with age.

### 4.1. Arrhythmias in Children Without CHDs

Sinus arrhythmia, premature atrial and ventricular contractions, and junctional rhythms occur frequently in neonates and are usually benign [72]. In a study of 360 healthy children, ranging from birth to adolescence, sinus arrhythmia was documented in all participants and premature atrial contractions were detected on Holter ECGs in 51% of healthy newborns [73]. Sinus arrhythmia and ectopy in children have been attributed to a higher vagal tone, an immature autonomic nervous system regulation, and CCS [74,75]. Southall et al. also reported a 14% incidence of supraventricular premature contractions among healthy neonates in the first 10 days of life [76]. In general, SVT is the most common symptomatic arrhythmia in infants and children, affecting approximately 1 in 250–1000 individuals [77]. Its incidence peaks within the first 2 months of life and often resolves during infancy, although recurrences may occur in some children between 6 and 8 years of age [77].

Atrioventricular reentrant tachycardia (AVRT), with an incidence of 16.3 per 100,000 live births, is the predominant neonatal tachyarrhythmia [78]. It is caused by persistence of an embryological accessory pathway that normally regresses before birth [79]. With aging, dual AV nodal physiology develops, with functional separation into fast and slow pathways [80]. AVNRT becomes more prevalent, especially among females [77,81].

Other forms of SVT occur less frequently. Focal atrial tachycardia originates from a small, circumscribed area, from where it expands centrifugally to the remainder of the atria. This arrhythmia accounts for 5% to 10% of all SVT cases during infancy [82]. Atrial flutter (AFL) is uncommon and most frequently seen in the neonatal period [81], with an incidence of 2.1 cases per 100,000 live births [78]. AF is extremely rare in otherwise healthy children and typically arises from degeneration of regular SVTs, such as AVRT or AVNRT [78].

Ventricular tachycardia (VT) is rare in pediatric patients with structurally normal hearts and accounts for approximately only 1.8% of pediatric electrophysiology studies [81,83]. It occurs most commonly in infancy; 86% of VTs originate from the RV, and up to 89% resolve spontaneously [81].

All degrees of AVB can occur in pediatric patients [77]. The prevalence of complete AVB is approximately 2.1 per 100,000 live births [78], which may result from auto-immune or inflammatory diseases [77]. In children requiring chronic ventricular pacing for high-degree AVB, epicardial LV apical/mid-lateral pacing better preserves LV synchrony and function than RV apical/free-wall pacing and is therefore preferred [64,65,66,67,68,69,70].

In addition, in structurally normal hearts, autonomic instability and immaturity of the CCS may play a role in the pathophysiology of SIDS. Postmortem studies have reported anomalies such as exaggerated resorptive degeneration at the AV junction, hypoplasia of nodal tissue, persistence of accessory AV connections, and displacement of the His bundle to the left side [84,85,86]. Such anomalies may increase electrical instability in early infancy, predisposing children to fatal arrhythmias.

### 4.2. Preoperative Arrhythmias in Children with CHDs

Most pediatric patients with CHD undergo surgical correction early in life, often before arrhythmias become clinically evident. Consequently, the natural progression of arrhythmogenesis in this young population is interrupted by early intervention. Patients with ASDs may remain uncorrected until adulthood and are more likely to develop arrhythmias earlier and more frequently than the general population [87]. However, in unrepaired tetralogy of Fallot (TOF), ventricular arrhythmias (VAs) may already appear during childhood [88]. In a study performed in 1984, VAs were defined as frequent premature ventricular contractions (PVCs) and/or short runs of non-sustained VT. The reported incidence of VAs was 20% (1/5) in patients aged 8–15 years and 58% (11/19) in those older than 16 years (~46 years old) [88]. However, studies reporting on the incidence of VAs in pediatric patients with unrepaired TOF are lacking. In future studies, more accurate definition of VAs would be helpful. For example, PVC burden should be reported as the percentage of beats, couplets and triplets should be listed separately, and clear criteria for non-sustained and sustained VT should be specified, with systematic recording of symptoms and management. Long-term noninvasive monitoring would be useful to optimize early intervention and risk stratification.

In addition to structural and/or hemodynamic abnormalities, patients with CHD are inherently more arrhythmogenic due to structural abnormalities of the CCS, as summarized in Table 1.

### 4.3. Postoperative Arrhythmias in Children with CHDs

In pediatric CHD surgery, intraoperative and postoperative arrhythmias are common but have distinct clinical implications. Intraoperative arrhythmias, such as second degree AVB and premature beats, are usually transient and unrelated to postoperative arrhythmias [89]. However, postoperative arrhythmias remain a major cause of morbidity and mortality in children and young adults with CHD [90]. Early postoperative arrhythmias (hours to days after surgery) are generally related to perioperative factors, such as younger age, low body weight, prolonged bypass time, and higher surgical complexity [91,92,93,94]. While typically transient and manageable with pharmacological therapy, these arrhythmias can significantly impact clinical outcomes, particularly in patients who are hemodynamically unstable [94,95]. In contrast, late arrhythmias (years after surgery) usually arise from structural remodeling, including fibrosis, dilatation, surgical scars, and prosthetic patches [96].

#### 4.3.1. Tachyarrhythmia

Junctional ectopic tachycardia (JET) and ectopic atrial tachycardia (EAT) are common early postoperative arrhythmias following CHD surgery, with reported incidences of 1.4–11.9% for JET [91,92,93,94,97,98,99,100,101,102,103] and 2.5–8% for EAT [104,105,106]. JET primarily affects neonates and young infants, typically arises within 72 h postoperatively [103], and resolves within 2–8 days in patients who are hemodynamically stable [100].

**Figure 3:**
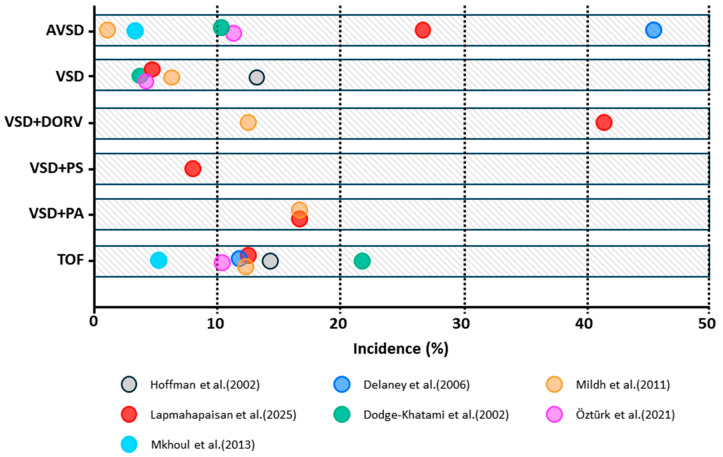
Bar graphs illustrating the reported incidence of postoperative junctional ectopic tachycardia (JET) across various CHD subtypes. AVSD: atrioventricular septal defect; CHD: congenital heart disease; DORV: double outlet right ventricle; PA: pulmonary atresia; PS: pulmonary stenosis; TOF: tetralogy of Fallot; VSD: ventricular septal defect [91,92,94,97,99,101,102].

JET typically occurs after surgical procedures involving the region of the His bundle, such as the repair of TOF, VSD, and AVSD, and is characterized by abnormal automaticity near the AVN with AV dissociation [103]. In contrast, EAT generally presents later, often within 14 days postoperatively [104,105,106]. Both arrhythmias are consistently associated with prolonged intensive care unit stays and increased duration of mechanical ventilation [97,98,101,102,104]. Although most studies have not demonstrated an increased mortality associated with JET, a cohort study of 874 patients reported a significant association between JET and higher mortality [98]. Figure 3 provides a summary of JET incidence across different CHD subtypes.

AFL and intra-atrial reentrant tachycardia (IART) are common postoperative reentrant arrhythmias, particularly in older children and adolescents following Fontan completion. In a multicenter study involving 520 children with a Fontan circulation, IART was reported in 7.3% of them; the risk of IART was highest during the first 2 years after Fontan completion, decreased between years 4 and 6, and then increased again in later childhood [107]. This prevalence is lower than the prevalence reported in 5-year follow-up studies performed in the last few decades, ranging from 16 to 22% [108,109,110]. Reentry circuits typically involve the cavotricuspid isthmus, atriotomy-related scar tissue, or patchy areas of scar tissue [111]. In a cohort of pediatric patients with CHD undergoing catheter ablation, the majority of AFL and IART circuits were localized to the cavotricuspid isthmus or areas of atrial scar [112].

Postoperative AF is rare among pediatric patients with CHD of all ages, with a reported incidence of <1% in three studies including 670, 402, and 262 patients, respectively [93,94,113]. This low prevalence may reflect the relative resistance of the pediatric atrial myocardium to hemodynamic stress during the early postoperative period [96,114]. However, AF becomes increasingly prevalent with age in patients with CHD who have undergone surgical intervention [13].

Similarly, VT is an uncommon postoperative tachyarrhythmia across any pediatric age, with reported incidences of ≤2% [91,92,93,94,115]. However, recent cohort studies suggest that VT may be more common than previously reported. In 886 pediatric patients with CHD, VTs occurred in the early postoperative period in 16% of the population [116]. This higher incidence likely reflects the continuous full-disclosure telemetry and inclusion of non-sustained VA episodes, whereas other studies primarily reported only clinically significant VAs.

The prevalence of VAs (not further specified) in a cohort of 2503 pediatric patients with CHD was 18.5%, requiring treatment in 29% of cases [117]. Monomorphic VT was the predominant subtype, comprising 62.3% of all VAs receiving treatment [117].

#### 4.3.2. Bradyarrhythmia

Bradyarrhythmia may result from direct surgical injury to the SAN or AVN.

SND occurs in approximately 8% (26/310) of pediatric patients after CHD surgery, particularly following atrial procedures (23/26) [115]. Its incidence is higher in patients who have undergone the Fontan procedure, with reported rates of 47.4% (46/97) and 29% (33/115) [118,119]. SND typically manifests as postoperative sinus bradycardia, often requiring temporary pacing, and may necessitate permanent pacemaker implantation in severe or persistent cases [115,118,119]. Postoperative complete AVB occurs in 1.5–3.7% of patients [91,93,94], generally secondary to intraoperative injury to the His–Purkinje system during VSD closure, subaortic resection, or myectomy [91,93,113]. While most cases resolve within 9 days, those persisting beyond 30 days rarely resolve and often require permanent pacemaker implantation [120]. The American Heart Association recommends permanent pacing for advanced second- or third-degree AVB persisting ≥ 7 days postoperatively (Class I indication) [121]. Delayed-onset complete AVB has also been reported months to years after surgery, especially following VSD closure, with all affected patients ultimately requiring pacemaker implantation [122,123,124].

## 5. Conclusions

This review highlights the complex interplay between postnatal cardiac development, congenital heart defects, and arrhythmogenesis, emphasizing the significance of inherent electrophysiological abnormalities, including CCS abnormalities, in determining arrhythmia vulnerability in patients with CHD.

Most reports primarily focus on arrhythmogenesis in adult CHD populations. At present, there is accumulating evidence suggesting that in patients with CHD, electrophysiological alterations are present early in life, even before corrective or palliative surgery.

However, studies exploring preoperative electrophysiology in pediatric CHD are scarce, and existing studies are constrained by small sample sizes, the lack of age-matched controls, and limited access to human tissue. In addition, there is a wide variation in reported endpoints across different studies. Differences in study design, patient selection, surgical techniques across various eras and regions, and the way arrhythmias are defined or monitored all contribute to this heterogeneity, making direct comparison across studies difficult. Despite these challenges, early electrical alterations appear to play an important role in later arrhythmias in pediatric patients with CHD, indicating the need for in-depth investigation of cardiac development and arrhythmogenesis in this population.

## 6. Future Directions and Clinical Implications

The wide endpoint variation in existing data complicates comparison between different studies. This inconsistency emphasizes the need for standardized criteria for patient selection, surgical approach, arrhythmia definitions, and monitoring strategies, with age- and/or sex-specific parameters. Multicenter studies, including large cohorts and longitudinal follow-up, are necessary. Recordings should be archived in a time-stamped clinical database (diagnosis, medication, procedure, perioperative events, and follow-up details) to support cross-center use. Standard surface ECGs and Holter recordings remain fundamental for follow-up due to their noninvasive nature, wide availability, and cross-center interpretation. Wearable devices extend monitoring into daily life, providing clinically useful data across ages and perioperative stages, especially during long-term follow-up under different conditions (rest, sleep, exercise).

In children without heart disease, noninvasive daily-life monitoring with wearable devices may identify higher risk patients (e.g., prolonged QTc in infants evaluated for SIDS) and inform timely evaluation, family screening, and indicated intervention.

In children with CHD, monitoring in the early postoperative period can be performed by combining continuous ECG registrations with bedside-amplified atrial electrograms recorded from temporary wires (e.g., AtriAmp), enabling early recognition of JET and novel AVB. Additionally, pacing can be performed without requiring additional interventions. During long-term follow-up, noninvasive tools, such as long-term continuous rhythm recordings by, e.g., wearable devices, enable ongoing rhythm surveillance and early detection of arrhythmias. This is especially important in CHDs due to their onset of arrhythmias at a young age. Moreover, electrical recordings obtained with noninvasive tools can be compared with recordings from healthy children, allowing us to distinguish disease-related electrical alterations from normal developmental changes.

However, early electrophysiological alterations may remain undetected in the surface ECG, which highlights the importance of high-resolution mapping of the endo- or epicardial surface.

As mentioned earlier, limitations in high-resolution epicardial mapping in humans underline the necessity of animal models. Animal models enable direct correlation of high-resolution maps with histological analysis to validate whether electrophysiological alterations (conduction block, low-voltage areas, focal activity, and large repolarization dispersion) are associated with structural changes such as fibrosis, abnormal fiber orientation, fibro-fatty replacement, changes in connexin expression, and localization. Animal models with pressure or volume overload and hypoxia can reproduce the pathophysiological conditions of CHD and allow investigation of their effects on cardiac electrophysiology by comparison with matched healthy controls. Surgical reconstructions that simulate CHD repairs enable repeated measurements before and after repair in the same animal. Early and long-term remapping after surgery, combined with histology, allows characterization of postoperative adaptation of cardiac electrophysiology. Moreover, small animal models, such as mice and rats, provide additional advantages: (1) they are less expensive, (2) they involve larger study numbers, and (3) they allow genetic manipulation. These models make them suitable for investigating the role of specific ion channel genes in cardiac development.

Conduction system mapping can provide data on AVN–His–Purkinje activation and may offer mechanistic insight into CHDs with CCS abnormalities (AVSD, perimembranous VSD, and ccTGA). Brief conduction system recordings intraoperatively can delineate AV conduction pathways and guide suture or patch placement without a separate procedure. Intraoperative His bundle mapping is associated with lower rates of postoperative AVB and pacemaker implantation in patients with heterotaxy syndrome and non-L-malposed great arteries [125].

Combining traditional tools (surface ECG, Holter recording) with new approaches (high-resolution mapping, conduction-system recording, wearable devices) can clarify (1) how electrophysiology evolves with development, (2) differences among various CHD subtypes, and (3) adaptation after surgery.

When an arrhythmia is noninvasively detected, further evaluation is needed to assess whether a patient is at high risk and early anti-arrhythmic therapy is mandatory or when close observation suffices. In the latter case, noninvasive monitoring can be used to evaluate whether there is spontaneous resolution or progression of the arrhythmia. High-resolution and conduction system mapping during surgery may provide mechanistic insights into arrhythmogenesis.

Combining noninvasive recordings with intraoperative mapping data may deepen our understanding of (CHD-related) arrhythmias, thereby improving risk stratification, optimizing surgical timing, and enhancing overall long-term outcomes for pediatric patients with CHD.

## Figures and Tables

**Table 1:** CHD associated with conduction system abnormalities.

CHD	Conduction System Abnormalities	Arrhythmias	References
ASD	Primum: posteroinferior displacement of the AVN and His bundle Secundum: AV nodal fast pathway Sinus venosus: close proximity to the SAN	AVB, SND AVNRT, SND SND	[7,51]
pmVSD	Posteroinferior displacement of the AVN and His bundle	AVB	[52,53]
AVSD	Posteroinferior displacement of the AVN and His bundle	AVB	[51,54,55]
ccTGA	Displacement of AVN, altered conduction axis	Complete AVB	[56,57]
Single ventricle	Displacement of AV conduction pathways	AVNRT	[55]
Left atrial isomerism	Hypoplastic SAN and malformed AVN	AVB; SND	[58,59]
Right atrial somerism	Dual SAN and dual AVN, potential for twin AVN pathways	AFL, SVTs, and VT	[58,59]
Ebstein’s anomaly	Compressed AVN, malformed right bundle branch, accessory pathways	AVB, tachyarrhythmias	[60]

## Data Availability

No new data were created or analyzed in this study. Data sharing is not applicable to this article.

## References

[1] Shekhar S., Agrawal A., Pampori A., Lak H., Windsor J., Ramakrishna H. (2022). Mortality in Adult Congenital Heart Disease: Analysis of Outcomes and Risk Stratification. J. Cardiothorac. Vasc. Anesth..

[2] van Zyl M., Kapa S., Padmanabhan D., Chen F.C., Mulpuru S.K., Packer D.L., Munger T.M., Asirvatham S.J., McLeod C.J. (2016). Mechanism and Outcomes of Catheter Ablation for Ventricular Tachycardia in Adults with Repaired Congenital Heart Disease. Heart Rhythm.

[3] Hoffman J.I.E., Kaplan S. (2002). The Incidence of Congenital Heart Disease. J. Am. Coll. Cardiol..

[4] Liu A., Diller G.-P., Moons P., Daniels C.J., Jenkins K.J., Marelli A. (2023). Changing Epidemiology of Congenital Heart Disease: Effect on Outcomes and Quality of Care in Adults. Nat. Rev. Cardiol..

[5] Zimmerman M.S., Smith A.G.C., Sable C.A., Echko M.M., Wilner L.B., Olsen H.E., Atalay H.T., Awasthi A., Bhutta Z.A., Boucher J.L. (2020). Global, Regional, and National Burden of Congenital Heart Disease, 1990–2017: A Systematic Analysis for the Global Burden of Disease Study 2017. Lancet Child Adolesc. Health.

[6] van der Bom T., Zomer A.C., Zwinderman A.H., Meijboom F.J., Bouma B.J., Mulder B.J.M. (2011). The Changing Epidemiology of Congenital Heart Disease. Nat. Rev. Cardiol..

[7] Kline J., Costantini O. (2019). Arrhythmias in Congenital Heart Disease. Med. Clin. N. Am..

[8] Salameh S., Ogueri V., Posnack N.G. (2023). Adapting to a New Environment: Postnatal Maturation of the Human Cardiomyocyte. J. Physiol..

[9] Wu M.-H., Chiu S.-N., Tseng W.-C., Lu C.-W., Kao F.-Y., Huang S.-K. (2023). Atrial Fibrillation in Adult Congenital Heart Disease and the General Population. Heart Rhythm.

[10] Kharbanda R.K., van Schie M.S., Ramdat Misier N.L., van Leeuwen W.J., Taverne Y.J.H.J., van de Woestijne P.C., Kammeraad J.A.E., Bartelds B., Bogers A.J.J.C., de Groot N.M.S. (2020). First Evidence of Atrial Conduction Disorders in Pediatric Patients With Congenital Heart Disease. JACC Clin. Electrophysiol..

[11] Kharbanda R.K., van Schie M.S., van Leeuwen W.J., Taverne Y.J.H.J., Houck C.A., Kammeraad J.A.E., Bogers A.J.J.C., de Groot N.M.S. (2021). First-in-Children Epicardial Mapping of the Heart: Unravelling Arrhythmogenesis in Congenital Heart Disease. Interact. Cardiovasc. Thorac. Surg..

[12] Dai L., Zhang C., Freriks A.I., Zheng J., Linderhof M.H.C., Nguyen H.H., van Schie M.S., Yildirim V., Knops P., Misier N.R. (2025). Small Patients, Significant Findings: Electrophysiological Properties of Bachmann’s Bundle in Pediatric Patients. Heart Rhythm.

[13] Mandalenakis Z., Rosengren A., Lappas G., Eriksson P., Gilljam T., Hansson P.-O., Skoglund K., Fedchenko M., Dellborg M. (2018). Atrial Fibrillation Burden in Young Patients With Congenital Heart Disease. Circulation.

[14] Martín de Miguel I., Ávila P. (2021). Atrial Fibrillation in Congenital Heart Disease. Eur. Cardiol. Rev..

[15] Kharbanda R.K., van Schie M.S., Ramdat Misier N.L., Wesselius F.J., Zwijnenburg R.D., van Leeuwen W.J., van de Woestijne P.C., de Jong P.L., Bogers A.J.J.C., Taverne Y.J.H.J. (2022). In-Vivo Sino-Atrial Node Mapping in Children and Adults with Congenital Heart Disease. Front. Pediatr..

[16] Walsh E.P., Cecchin F. (2007). Arrhythmias in Adult Patients With Congenital Heart Disease. Circulation.

[17] Miquerol L., Beyer S., Kelly R.G. (2011). Establishment of the Mouse Ventricular Conduction System. Cardiovasc. Res..

[18] Bhattacharyya S., Munshi N.V. (2020). Development of the Cardiac Conduction System. Cold Spring Harb. Perspect. Biol..

[19] James T.N. (2002). Structure and Function of the Sinus Node, AV Node and His Bundle of the Human Heart: Part I—Structure. Prog. Cardiovasc. Dis..

[20] James T.N. (2003). Structure and Function of the Sinus Node, AV Node and His Bundle of the Human Heart: Part II—Function. Prog. Cardiovasc. Dis..

[21] Postma A.V., Christoffels V.M., Bezzina C.R. (2011). Developmental Aspects of Cardiac Arrhythmogenesis. Cardiovasc. Res..

[22] van Weerd J.H., Christoffels V.M. (2016). The Formation and Function of the Cardiac Conduction System. Development.

[23] Christoffels V.M., Smits G.J., Kispert A., Moorman A.F.M. (2010). Development of the Pacemaker Tissues of the Heart. Circ. Res..

[24] Stroud D.M., Gaussin V., Burch J.B.E., Yu C., Mishina Y., Schneider M.D., Fishman G.I., Morley G.E. (2007). Abnormal Conduction and Morphology in the Atrioventricular Node of Mice with Atrioventricular Canal–Targeted Deletion of Alk3/Bmpr1a Receptor. Circulation.

[25] Feulner L., van Vliet P.P., Puceat M., Andelfinger G. (2022). Endocardial Regulation of Cardiac Development. J. Cardiovasc. Dev. Dis..

[26] Keith A., Flack Martin W. (1906). The Auriculo-Ventricular Bundle of the Human Heart. Lancet.

[27] James T.N. (1968). Sudden Death in Babies: New Observations in the Heart. Am. J. Cardiol..

[28] Nosetti L., Zaffanello M., Lombardi C., Gerosa A., Piacentini G., Abramo M., Agosti M. (2024). Early Screening for Long QT Syndrome and Cardiac Anomalies in Infants: A Comprehensive Study. Clin. Pract..

[29] Cazzato F., Coll M., Grassi S., Fernàndez-Falgueras A., Nogué-Navarro L., Iglesias A., Castellà J., Oliva A., Brugada R. (2024). Investigating Cardiac Genetic Background in Sudden Infant Death Syndrome (SIDS). Int. J. Legal Med..

[30] Millat G., Kugener B., Chevalier P., Chahine M., Huang H., Malicier D., Rodriguez-Lafrasse C., Rousson R. (2009). Contribution of Long-QT Syndrome Genetic Variants in Sudden Infant Death Syndrome. Pediatr. Cardiol..

[31] Arnestad M., Crotti L., Rognum T.O., Insolia R., Pedrazzini M., Ferrandi C., Vege A., Wang D.W., Rhodes T.E., George A.L. (2007). Prevalence of Long-QT Syndrome Gene Variants in Sudden Infant Death Syndrome. Circulation.

[32] Liu J., Laksman Z., Backx P.H. (2016). The Electrophysiological Development of Cardiomyocytes. Adv. Drug Deliv. Rev..

[33] Karbassi E., Fenix A., Marchiano S., Muraoka N., Nakamura K., Yang X., Murry C.E. (2020). Cardiomyocyte Maturation: Advances in Knowledge and Implications for Regenerative Medicine. Nat. Rev. Cardiol..

[34] Wang L., Duff H.J. (1997). Developmental Changes in Transient Outward Current in Mouse Ventricle. Circ. Res..

[35] Trépanier-Boulay V., Lupien M.-A., St-Michel C., Fiset C. (2004). Postnatal Development of Atrial Repolarization in the Mouse. Cardiovasc. Res..

[36] Kilborn M.J., Fedida D. (1990). A Study of the Developmental Changes in Outward Currents of Rat Ventricular Myocytes. J. Physiol..

[37] Xu H., Dixon J.E., Barry D.M., Trimmer J.S., Merlie J.P., McKinnon D., Nerbonne J.M. (1996). Developmental Analysis Reveals Mismatches in the Expression of K+ Channel Alpha Subunits and Voltage-Gated K+ Channel Currents in Rat Ventricular Myocytes. J. Gen. Physiol..

[38] Guo W., Kamiya K., Toyama J. (1996). Modulated Expression of Transient Outward Current in Cultured Neonatal Rat Ventricular Myocytes: Comparison with Development in Situ. Cardiovasc. Res..

[39] Wahler G.M., Dollinger S.J., Smith J.M., Flemal K.L. (1994). Time Course of Postnatal Changes in Rat Heart Action Potential and in Transient Outward Current Is Different. Am. J. Physiol..

[40] Shimoni Y., Fiset C., Clark R.B., Dixon J.E., McKinnon D., Giles W.R. (1997). Thyroid Hormone Regulates Postnatal Expression of Transient K+ Channel Isoforms in Rat Ventricle. J. Physiol..

[41] Leuranguer V., Monteil A., Bourinet E., Dayanithi G., Nargeot J. (2000). T-Type Calcium Currents in Rat Cardiomyocytes during Postnatal Development: Contribution to Hormone Secretion. Am. J. Physiol. Heart Circ. Physiol..

[42] Wang Y., Xu H., Kumar R., Tipparaju S.M., Wagner M.B., Joyner R.W. (2003). Differences in Transient Outward Current Properties between Neonatal and Adult Human Atrial Myocytes. J. Mol. Cell. Cardiol..

[43] Roca T.P., Pigott J.D., Clarkson C.W., Crumb W.J. (1996). L-Type Calcium Current in Pediatric and Adult Human Atrial Myocytes: Evidence for Developmental Changes in Channel Inactivation. Pediatr. Res..

[44] Escande D., Loisance D., Planche C., Coraboeuf E. (1985). Age-Related Changes of Action Potential Plateau Shape in Isolated Human Atrial Fibers. Am. J. Physiol.-Heart Circ. Physiol..

[45] Semizel E., Öztürk B., Bostan O.M., Cil E., Ediz B. (2008). The Effect of Age and Gender on the Electrocardiogram in Children. Cardiol. Young.

[46] Lue H.-C., Wu M.-H., Wang J.-K., Lin M.-T., Lu C.-W., Chiu S.-N., Chen C.-A., Wu E.-T., Wang C.-C., Fu C.-M. (2023). Normal ECG Standards and Percentile Charts for Infants, Children and Adolescents. Pediatr. Neonatol..

[47] Bratincsák A., Kimata C., Limm-Chan B.N., Vincent K.P., Williams M.R., Perry J.C. (2020). Electrocardiogram Standards for Children and Young Adults Using Z-Scores. Circ. Arrhythm. Electrophysiol..

[48] Zubrzycki M., Schramm R., Costard-Jäckle A., Grohmann J., Gummert J.F., Zubrzycka M. (2024). Cardiac Development and Factors Influencing the Development of Congenital Heart Defects (CHDs): Part I. Int. J. Mol. Sci..

[49] Jongbloed M.R.M., Vicente Steijn R., Hahurij N.D., Kelder T.P., Schalij M.J., Gittenberger-de Groot A.C., Blom N.A. (2012). Normal and Abnormal Development of the Cardiac Conduction System; Implications for Conduction and Rhythm Disorders in the Child and Adult. Differentiation.

[50] Jongbloed M.R.M., Mahtab E.a.F., Blom N.A., Schalij M.J., Gittenberger-de Groot A.C. (2008). Development of the Cardiac Conduction System and the Possible Relation to Predilection Sites of Arrhythmogenesis. Sci. World J..

[51] Williams M.R., Perry J.C. (2018). Arrhythmias and Conduction Disorders Associated with Atrial Septal Defects. J. Thorac. Dis..

[52] Lin L., Liu J., Guo X., Chen H., Huang Y., Zheng H., Chen W., Chen L., Chen L., Chen Z. (2022). Risk Factors for Atrioventricular Block after Occlusion for Perimembranous Ventricular Septal Defect. Heart Rhythm.

[53] Feins E.N., Nido P.J. (2023). del Conduction in Congenital Heart Surgery. J. Thorac. Cardiovasc. Surg..

[54] Di Mambro C., Calvieri C., Silvetti M.S., Tamburri I., Giannico S., Baban A., Albanese S., Brancaccio G., Carotti A., Iorio F.S. (2018). Bradyarrhythmias in Repaired Atrioventricular Septal Defects: Single-Center Experience Based on 34 Years of Follow-up of 522 Patients. Pediatr. Cardiol..

[55] Waldmann V., Hebe J., Walsh E.P., Khairy P., Ernst S. (2022). Catheter Ablation of Atrioventricular Nodal Reentrant Tachycardia in Patients with Congenital Heart Disease. Circ. Arrhythm. Electrophysiol..

[56] Wallis G.A., Debich-Spicer D., Anderson R.H. (2011). Congenitally Corrected Transposition. Orphanet J. Rare Dis..

[57] Cardell B.S. (1956). Corrected Transposition of the Great Vessels. Br. Heart J..

[58] Ozawa Y., Asakai H., Shiraga K., Shindo T., Hirata Y., Hirata Y., Inuzuka R. (2019). Cardiac Rhythm Disturbances in Heterotaxy Syndrome. Pediatr. Cardiol..

[59] Loomba R.S., Willes R.J., Kovach J.R., Anderson R.H. (2016). Chronic Arrhythmias in the Setting of Heterotaxy: Differences between Right and Left Isomerism. Congenit. Heart Dis..

[60] Attenhofer Jost C.H., Connolly H.M., Dearani J.A., Edwards W.D., Danielson G.K. (2007). Ebstein’s Anomaly. Circulation.

[61] Baruteau A., Abrams D.J., Ho S.Y., Thambo J., McLeod C.J., Shah M.J. (2017). Cardiac Conduction System in Congenitally Corrected Transposition of the Great Arteries and Its Clinical Relevance. J. Am. Heart Assoc. Cardiovasc. Cerebrovasc. Dis..

[62] Silvetti M.S., Favoccia C., Saputo F.A., Tamburri I., Mizzon C., Campisi M., Gimigliano F., Rinelli G., Rava L., Drago F. (2023). Three-Dimensional-Mapping-Guided Permanent Conduction System Pacing in Paediatric Patients with Congenitally Corrected Transposition of the Great Arteries. Europace.

[63] Huhta J., Maloney J., Ritter D., Ilstrup D., Feldt R. (1983). Complete Atrioventricular Block in Patients with Atrioventricular Discordance. Circulation.

[64] Shah M.J., Silka M.J., Avari Silva J.N., Balaji S., Beach C.M., Benjamin M.N., Berul C.I., Cannon B., Cecchin F., Cohen M.I. (2021). 2021 PACES Expert Consensus Statement on the Indications and Management of Cardiovascular Implantable Electronic Devices in Pediatric Patients. Indian Pacing Electrophysiol. J..

[65] van Geldorp I.E., Vanagt W.Y., Bauersfeld U., Tomaske M., Prinzen F.W., Delhaas T. (2009). Chronic Left Ventricular Pacing Preserves Left Ventricular Function in Children. Pediatr. Cardiol..

[66] Gebauer R.A., Tomek V., Kubus P., Rázek V., Matejka T., Salameh A., Kostelka M., Janousek J. (2009). Differential Effects of the Site of Permanent Epicardial Pacing on Left Ventricular Synchrony and Function in the Young: Implications for Lead Placement. EP Eur..

[67] van Geldorp I.E., Delhaas T., Gebauer R.A., Frias P., Tomaske M., Friedberg M.K., Tisma-Dupanovic S., Elders J., Früh A., Gabbarini F. (2011). Impact of the Permanent Ventricular Pacing Site on Left Ventricular Function in Children: A Retrospective Multicentre Survey. Heart Br. Card. Soc..

[68] Janoušek J., van Geldorp I.E., Krupičková S., Rosenthal E., Nugent K., Tomaske M., Früh A., Elders J., Hiippala A., Kerst G. (2013). Permanent Cardiac Pacing in Children: Choosing the Optimal Pacing Site. Circulation.

[69] Tomaske M., Breithardt O.A., Bauersfeld U. (2009). Preserved Cardiac Synchrony and Function with Single-Site Left Ventricular Epicardial Pacing during Mid-Term Follow-up in Paediatric Patients. EP Eur..

[70] Salameh A., Dhein S., Blanke K., Rastan A., Hiyasat B., Dietze A., Sobiraij A., Dähnert I., Janousek J. (2012). Right or Left Ventricular Pacing in Young Minipigs with Chronic Atrioventricular Block: Long-Term in Vivo Cardiac Performance, Morphology, Electrophysiology, and Cellular Biology. Circulation.

[71] Khairy P. (2016). Ventricular Arrhythmias and Sudden Cardiac Death in Adults with Congenital Heart Disease. Heart.

[72] Ban J.-E. (2017). Neonatal Arrhythmias: Diagnosis, Treatment, and Clinical Outcome. Korean J. Pediatr..

[73] Nagashima M., Matsushima M., Ogawa A., Ohsuga A., Kaneko T., Yazaki T., Okajima M. (1987). Cardiac Arrhythmias in Healthy Children Revealed by 24-Hour Ambulatory ECG Monitoring. Pediatr. Cardiol..

[74] Lubocka P., Sabiniewicz R. (2021). Respiratory Sinus Arrhythmia in Children—Predictable or Random?. Front. Cardiovasc. Med..

[75] Azak E., Cetin I.I. (2021). Premature Cardiac Beats in Children with Structurally Normal Heart: Autonomic Dysregulation. Pediatr. Int..

[76] Southall D.P., Richards J., Mitchell P., Brown D.J., Johnston P.G., Shinebourne E.A. (1980). Study of Cardiac Rhythm in Healthy Newborn Infants. Br. Heart J..

[77] Sharieff G.Q., Rao S.O. (2006). The Pediatric ECG. Emerg. Med. Clin. N. Am..

[78] Turner C.J., Wren C. (2013). The Epidemiology of Arrhythmia in Infants: A Population-Based Study. J. Paediatr. Child Health.

[79] Srinivasan C., Balaji S. (2019). Neonatal Supraventricular Tachycardia. Indian Pacing Electrophysiol. J..

[80] Blaufox A.D., Rhodes J.F., Fishberger S.B. (2000). Age Related Changes in Dual AV Nodal Physiology. Pacing Clin. Electrophysiol..

[81] Brugada J., Blom N., Sarquella-Brugada G., Blomstrom-Lundqvist C., Deanfield J., Janousek J., Abrams D., Bauersfeld U., Brugada R., Drago F. (2013). Pharmacological and Non-Pharmacological Therapy for Arrhythmias in the Pediatric Population: EHRA and AEPC-Arrhythmia Working Group Joint Consensus Statement. EP Eur..

[82] Tasci O., Karadeniz C. (2023). Ivabradine in a 15-Day-Old Male Neonate with Refractory Focal Atrial Tachycardia. Pacing Clin. Electrophysiol..

[83] Van Hare G.F., Javitz H., Carmelli D., Saul J.P., Tanel R.E., Fischbach P.S., Kanter R.J., Schaffer M., Dunnigan A., Colan S. (2004). Prospective Assessment after Pediatric Cardiac Ablation: Demographics, Medical Profiles, and Initial Outcomes. J. Cardiovasc. Electrophysiol..

[84] Bharati S., Krongrad E., Lev M. (1985). Study of the Conduction System in a Population of Patients with Sudden Infant Death Syndrome. Pediatr. Cardiol..

[85] Ottaviani G., Buja L.M. (2016). Anatomopathological Changes of the Cardiac Conduction System in Sudden Cardiac Death, Particularly in Infants: Advances over the Last 25 Years. Cardiovasc. Pathol. Off. J. Soc. Cardiovasc. Pathol..

[86] Matturri L., Ottaviani G., Ramos S.G., Rossi L. (2000). Sudden Infant Death Syndrome (SIDS): A Study of Cardiac Conduction System. Cardiovasc. Pathol. Off. J. Soc. Cardiovasc. Pathol..

[87] Brida M., Chessa M., Celermajer D., Li W., Geva T., Khairy P., Griselli M., Baumgartner H., Gatzoulis M.A. (2022). Atrial Septal Defect in Adulthood: A New Paradigm for Congenital Heart Disease. Eur. Heart J..

[88] Deanfield J.E., McKenna W.J., Presbitero P., England D., Graham G.R., Hallidie-Smith K. (1984). Ventricular Arrhythmia in Unrepaired and Repaired Tetralogy of Fallot. Relation to Age, Timing of Repair, and Haemodynamic Status. Br. Heart J..

[89] Houck C.A., Ramdjan T.T.T.K., Yaksh A., Teuwen C.P., Lanters E.A.H., Bogers A.J.J.C., de Groot N.M.S. (2018). Intraoperative Arrhythmias in Children with Congenital Heart Disease: Transient, Innocent Events?. EP Eur..

[90] Khairy P., Van Hare G.F., Balaji S., Berul C.I., Cecchin F., Cohen M.I., Daniels C.J., Deal B.J., Dearani J.A., Groot N.D. (2014). PACES/HRS Expert Consensus Statement on the Recognition and Management of Arrhythmias in Adult Congenital Heart Disease. Can. J. Cardiol..

[91] Delaney J.W., Moltedo J.M., Dziura J.D., Kopf G.S., Snyder C.S. (2006). Early Postoperative Arrhythmias after Pediatric Cardiac Surgery. J. Thorac. Cardiovasc. Surg..

[92] Lapmahapaisan S., Sateantantikul N., Maisat W. (2025). Revisiting Risk Factors and Incidence of Postoperative Tachyarrhythmias in Pediatric Cardiac Surgery. Sci. Rep..

[93] Rękawek J., Kansy A., Miszczak-Knecht M., Manowska M., Bieganowska K., Brzezinska-Paszke M., Szymaniak E., Turska-Kmieć A., Maruszewski P., Burczyński P. (2007). Risk Factors for Cardiac Arrhythmias in Children with Congenital Heart Disease after Surgical Intervention in the Early Postoperative Period. J. Thorac. Cardiovasc. Surg..

[94] Öztürk E., Kafalı H.C., Tanıdır İ.C., Tunca Şahin G., Onan İ.S., Haydin S., Güzeltaş A., Ergül Y. (2021). Early Postoperative Arrhythmias in Patients Undergoing Congenital Heart Surgery. Turk. J. Thorac. Cardiovasc. Surg..

[95] Kerr S., O’Leary E., DeWitt E.S., Mah D.Y., Alexander M.E., Kheir J.N., Feins E.N., Walsh E.P., Triedman J.K., Dionne A. (2024). Efficacy and Safety of Early Postoperative Ablation in Patients with Congenital Heart Disease. Heart Rhythm.

[96] Joye R., Beghetti M., Wacker J., Malaspinas I., Bouhabib M., Polito A., Bordessoule A., Shah D.C. (2023). Early and Late Postoperative Tachyarrhythmias in Children and Young Adults Undergoing Congenital Heart Disease Surgery. Pediatr. Cardiol..

[97] Mildh L., Hiippala A., Rautiainen P., Pettilä V., Sairanen H., Happonen J.-M. (2011). Junctional Ectopic Tachycardia after Surgery for Congenital Heart Disease: Incidence, Risk Factors and Outcome. Eur. J. Cardiothorac. Surg..

[98] Andreasen J.B., Johnsen S.P., Ravn H.B. (2008). Junctional Ectopic Tachycardia after Surgery for Congenital Heart Disease in Children. Intensive Care Med..

[99] Hoffman T.M., Bush D.M., Wernovsky G., Cohen M.I., Wieand T.S., Gaynor J.W., Spray T.L., Rhodes L.A. (2002). Postoperative Junctional Ectopic Tachycardia in Children: Incidence, Risk Factors, and Treatment. Ann. Thorac. Surg..

[100] Dodge-Khatami A., Miller O.I., Anderson R.H., Goldman A.P., Gil-Jaurena J.M., Elliott M.J., Tsang V.T., De Leval M.R. (2002). Surgical Substrates of Postoperative Junctional Ectopic Tachycardia in Congenital Heart Defects. J. Thorac. Cardiovasc. Surg..

[101] Makhoul M., Oster M., Fischbach P., Das S., Deshpande S. (2013). Junctional Ectopic Tachycardia after Congenital Heart Surgery in the Current Surgical Era. Pediatr. Cardiol..

[102] Dodge-Khatami A., Miller O.I., Anderson R.H., Gil-Jaurena J.M., Goldman A.P., de Leval M.R. (2002). Impact of Junctional Ectopic Tachycardia on Postoperative Morbidity Following Repair of Congenital Heart Defects. Eur. J. Cardiothorac. Surg..

[103] Erickson S.J. (2006). Guidelines for the Management of Junctional Ectopic Tachycardia Following Cardiac Surgery in Children. Curr. Paediatr..

[104] Rosales A.M., Walsh E.P., Wessel D.L., Triedman J.K. (2001). Postoperative Ectopic Atrial Tachycardia in Children with Congenital Heart Disease. Am. J. Cardiol..

[105] Clark B.C., Berger J.T., Berul C.I., Jonas R.A., Kaltman J.R., Lapsa J., Nath D.S., Sherwin E.D., Sinha P., Zurakowski D. (2018). Risk Factors for Development of Ectopic Atrial Tachycardia in Post-Operative Congenital Heart Disease. Pediatr. Cardiol..

[106] Uniat J., Hill A.C., Shwayder M., Silka M.J., Bar-Cohen Y. (2023). Ectopic Atrial Tachycardia in Infants Following Congenital Heart Disease Surgery. Pediatr. Cardiol..

[107] Stephenson E.A., Lu M., Berul C.I., Etheridge S.P., Idriss S.F., Margossian R., Reed J.H., Prakash A., Sleeper L.A., Vetter V.L. (2010). Arrhythmias in a Contemporary Fontan Cohort: Prevalence and Clinical Associations in a Multicenter Cross-Sectional Study. J. Am. Coll. Cardiol..

[108] Durongpisitkul K., Porter C.J., Cetta F., Offord K.P., Slezak J.M., Puga F.J., Schaff H.V., Danielson G.K., Driscoll D.J. (1998). Predictors of Early- and Late-Onset Supraventricular Tachyarrhythmias after Fontan Operation. Circulation.

[109] Fishberger S.B., Wernovsky G., Gentles T.L., Gauvreau K., Burnett J., Mayer J.E., Walsh E.P. (1997). Factors That Influence the Development of Atrial Flutter after the Fontan Operation. J. Thorac. Cardiovasc. Surg..

[110] Gelatt M., Hamilton R.M., McCrindle B.W., Gow R.M., Williams W.G., Trusler G.A., Freedom R.M. (1994). Risk Factors for Atrial Tachyarrhythmias after the Fontan Operation. J. Am. Coll. Cardiol..

[111] Ganea G., Cinteză E.E., Filip C., Iancu M.A., Balta M.D., Vătășescu R., Vasile C.M., Cîrstoveanu C., Bălgrădean M. (2023). Postoperative Cardiac Arrhythmias in Pediatric and Neonatal Patients with Congenital Heart Disease—A Narrative Review. Life.

[112] Houck C.A., Chandler S.F., Bogers A.J.J.C., Triedman J.K., Walsh E.P., de Groot N.M.S., Abrams D.J. (2019). Arrhythmia Mechanisms and Outcomes of Ablation in Pediatric Patients With Congenital Heart Disease. Circ. Arrhythm. Electrophysiol..

[113] Hoffman T.M., Wernovsky G., Wieand T.S., Cohen M.I., Jennings A.C., Vetter V.L., Godinez R.I., Gaynor J.W., Spray T.L., Rhodes L.A. (2002). The Incidence of Arrhythmias in a Pediatric Cardiac Intensive Care Unit. Pediatr. Cardiol..

[114] Maisat W., Lapmahapaisan S. (2024). Early Postoperative Tachyarrhythmias in Adult Congenital Heart Surgery: An Eight-Year Review at a Tertiary University Hospital in Thailand. J. Thorac. Dis..

[115] Pfammatter J.-P., Bachmann D.C.G., Wagner B.P., Pavlovic M., Berdat P., Carrel T., Pfenninger J. (2001). Early Postoperative Arrhythmias after Open-Heart Procedures in Children with Congenital Heart Disease. Pediatr. Crit. Care Med..

[116] Smith A.H., Flack E.C., Borgman K.Y., Owen J.P., Fish F.A., Bichell D.P., Kannankeril P.J. (2014). A Common Angiotensin-Converting Enzyme (ACE) Polymorphism and Preoperative ACE Inhibition Modify Risk of Tachyarrhythmias after Congenital Heart Surgery. Heart Rhythm Off. J. Heart Rhythm Soc..

[117] Fuchs S.R., Smith A.H., Van Driest S.L., Crum K.F., Edwards T.L., Kannankeril P.J. (2019). Incidence and Effect of Early Postoperative Ventricular Arrhythmias after Congenital Heart Surgery. Heart Rhythm.

[118] Bossers S.S.M., Duppen N., Kapusta L., Maan A.C., Duim A.R., Bogers A.J.J.C., Hazekamp M.G., van Iperen G., Helbing W.A., Blom N.A. (2015). Comprehensive Rhythm Evaluation in a Large Contemporary Fontan Population. Eur. J. Cardiothorac. Surg..

[119] Manning P.B., Mayer J.E., Wernovsky G., Fishberger S.B., Walsh E.P. (1996). Staged Operation to Fontan Increases the Incidence of Sinoatrial Node Dysfunction. J. Thorac. Cardiovasc. Surg..

[120] Weindling S.N., Saul J.P., Gamble W.J., Mayer J.E., Wessel D., Walsh E.P. (1998). Duration of Complete Atrioventricular Block after Congenital Heart Disease Surgery. Am. J. Cardiol..

[121] Epstein A.E., DiMarco J.P., Ellenbogen K.A., Estes N.A.M., Freedman R.A., Gettes L.S., Gillinov A.M., Gregoratos G., Hammill S.C., Hayes D.L. (2008). ACC/AHA/HRS 2008 Guidelines for Device-Based Therapy of Cardiac Rhythm Abnormalities. J. Am. Coll. Cardiol..

[122] Liberman L., Pass R.H., Hordof A.J., Spotnitz H.M. (2008). Late Onset of Heart Block after Open Heart Surgery for Congenital Heart Disease. Pediatr. Cardiol..

[123] Butera G., Carminati M., Chessa M., Piazza L., Micheletti A., Negura D.G., Abella R., Giamberti A., Frigiola A. (2007). Transcatheter Closure of Perimembranous Ventricular Septal Defects: Early and Long-Term Results. J. Am. Coll. Cardiol..

[124] Predescu D., Chaturvedi R.R., Friedberg M.K., Benson L.N., Ozawa A., Lee K.-J. (2008). Complete Heart Block Associated with Device Closure of Perimembranous Ventricular Septal Defects. J. Thorac. Cardiovasc. Surg..

[125] O’Leary E.T., Feins E.N., Davee J., Baird C.W., Beroukhim R., del Nido P.J., Dionne A., Gauvreau K., Hoganson D.M., Triedman J.K. (2024). Intraoperative Conduction Mapping to Reduce Postoperative Atrioventricular Block in Complex Congenital Heart Disease. JACC.

